# Physical function versus cognitive status: Which better predicts independence in basic activities among long‐lived cognitively preserved older adults

**DOI:** 10.1111/ajag.70093

**Published:** 2025-09-15

**Authors:** Juliedy Waldow Kupske, Júlia de Mattos, Tainara Steffens, Eduarda Blanco‐Rambo, Marcelo Bandeira‐Guimarães, Mikel L. Sáez de Asteasu, Mikel Izquierdo, Eduardo Lusa Cadore, Caroline Pietta‐Dias

**Affiliations:** ^1^ Exercise Research Laboratory, School of Physical Education, Physiotherapy and Dance Universidade Federal do Rio Grande do Sul Porto Alegre Brazil; ^2^ Navarrabiomed Hospital Universitario de Navarra (HUN)‐Universidad Pública de Navarra (UPNA), IdiSNA Pamplona Spain; ^3^ CIBER of Frailty and Healthy Aging (CIBERFES) Instituto de Salud Carlos III Madrid Spain

**Keywords:** aging, cognition, functional status, oldest old

## Abstract

**Objectives:**

This study aimed to compare physical function and cognitive status according to the level of dependence in basic activities of daily living (ADL) and to determine which of these variables better predict functional independence.

**Methods:**

This cross‐sectional study included 231 individuals over 90 years of age. Physical function was assessed using the Timed Up and Go (TUG) test and handgrip strength (HGS), and cognitive function was assessed using the Mini‐Mental State Examination (MMSE). A binary logistic regression adjusted for age was conducted to identify which variables related to physical performance or cognitive status could predict independence in basic ADL.

**Results:**

The results showed that the model including TUG (OR = 4.3; 95% CI = 2.18–8.34) along with HGS (OR = 2.0; 95% CI = 1.14–3.45) provided the best fit. This indicates that better performance on the TUG test increases the odds of independence by 4.3 times, and each increment in HGS doubles that chance.

**Conclusions:**

We conclude that older individuals with better physical function and greater muscle strength are more likely to be independent in ADL.

**Policy impact**

This study highlights the need for promoting individualised physical exercise within health‐care institutions for the oldest old to preserve functional independence during ageing.

**Practice impact**

Health professionals should focus on interventions that improve physical function by enhancing muscle strength and gait ability. This approach increases the chances of maintaining functional independence among the oldest old.

## 1 INTRODUCTION

Accelerated population ageing represents one of the most significant challenges faced by most countries.^1^ The growing number of long‐lived older adults is particularly concerning, since the oldest old often present comorbidities and disabilities that require ongoing assistance and daily multiprofessional care.^2^, ^3^ One of the main challenges highlighted by the World Health Organization (WHO) with ageing is preserving functional capacity, especially regarding activities of daily living (ADL), which are categorised into basic and instrumental ADL.^4^

Basic ADL encompasses essential self‐care skills, such as ambulation, feeding, dressing, personal hygiene, continence and toileting,^3^, ^5^ and these functional abilities tend to decline with advancing age, especially among the oldest old. Therefore, ADL serves as an important marker of functional status and autonomy in this population.^6^

Functional decline, defined as the inability to perform basic or instrumental ADL, is associated with an increased risk of morbidity and mortality in older populations.^6^ The relationship between physical function, cognitive status and overall functionality has garnered significant interest. Moreover, poor performance on physical function tests is one of the criteria for identifying physical frailty and sarcopenia, and predisposes individuals to dependency.^7^, ^8^, ^9^ Slow gait speed and difficulty rising from a chair have been linked to disability in ADL, which in turn is associated with poorer quality of life and higher health‐care costs.^10^ Conversely, patterns of difficulty in ADL—especially the more complex ones—may be related to cognitive deterioration.^10^, ^11^ The causal relationship between cognitive impairment and functional decline seems to be bidirectional in older populations.^12^, ^13^

Although longitudinal data in long‐lived older individuals suggest an increase in dependence for basic ADL over time,^14^ it remains unclear which domains (physical or cognitive) are the most critical determinants for maintaining autonomy in advanced age. The literature lacks studies that comparatively investigate the role of these domains as objective predictors of functional independence, particularly in clinical and institutional contexts. This gap is significant because identifying the dominant predictor could redirect clinical strategies: if physical function is more determinant, resistance‐training interventions should be prioritised^15^; if cognition is more influential, multidomain programs combining physical exercise with neuropsychological rehabilitation, cognitive stimulation interventions and early dementia diagnosis become paramount.^16^

Thus, the primary aim of this study was to compare physical function and cognitive status according to the level of dependence on basic ADL. The secondary objective was to determine which parameters better predict functional independence in nonagenarian and centenarian adults. We hypothesised that more independent participants would have greater physical performance and cognitive status, and physical and cognitive function outcomes would be independent predictors of the ability to perform basic ADL.

## 2 METHODS

### 2.1 Participants

This study included participants recruited from Primary Health Care Units, retirees' associations, long‐term care institutions for older individuals (LTCIs), senior community centres and religious institutions. The eligibility criteria were as follows: age 90 years and older; Mini‐Mental State Examination (MMSE) score 19 or higher for those without formal education and 23 or higher for those with schooling; ability to ambulate; no use of a wheelchair; no diagnosis of Parkinson's disease; and no upper‐ or lower‐limb amputation. The sample size calculation was performed in G*Power version 3.1.9.4 for *z*‐tests and logistic regression, based on the physical activity variable with an odds ratio of .56 from Wu et al. (2017),^17^ adopting an effect size of .5, power of 95% and significance level of .05. This yielded the required sample of 208 participants.

The study protocol was approved by the Research Ethics Committee of the Federal University of Rio Grande do Sul (CAAE: 79748517.5.0000.5347), the Research Ethics Committee of the State Department of Health (CAAE: 79748517.5.3002.5312) and the Research Ethics Committee of the Municipal Health Department of the city of Porto Alegre (Brazil) (CAAE: 79748517.5.3001.5338). All participants or their legal guardians provided informed consent. Data collection took place in the participants' homes and long‐term care institutions.

### 2.2 Assessment protocols

Cognitive function was assessed by the MMSE.^18^ It comprises 11 open‐ended questions grouped into six categories: temporal and spatial orientation, processing, attention, calculation, evocation, language and constructive ability, with higher scores indicating better cognitive performance.

Independence in basic ADLs was evaluated using the Katz Index,^19^, ^20^ a validated instrument that measures the ability to perform six essential basic ADLs: bathing, dressing, toileting, transferring, continence and feeding. The index consisted of six items with three response options, each corresponding to the level of assistance required. The total score from the items resulted in one of three classifications: independent (6 points), partially dependent (4–5 points) or dependent (0–3 points).

Physical function was assessed using the Timed Up and Go (TUG) test performed at habitual velocity, in which the participant rose from a chair, walked 3 metres at a usual pace, turned around, returned to the chair and sat down. Two trials were performed; the time for each trial (in seconds) was recorded, and the best (shortest) time was used for analysis.

Handgrip strength (HGS) was measured using a Jamar Hydraulic Hand Dynamometer (SH5001) (Saehan, Londerzeel, Belgium). The test was administered with the participant seated, shoulder adducted, elbow flexed at 90°, forearm in a neutral position and wrist between 0° and 30° of extension. Following the examiner's loud verbal ‘start’ command, the participant gripped the dynamometer with their dominant hand, held the contraction for 3 s and then relaxed. Two trials were recorded, and the average kilogram force (kgf) was used in the analysis.^21^

### 2.3 Statistical analysis

Descriptive statistics (means, standard deviations, medians and frequencies) were calculated. For the fundamental ADL variable analysis, participants were divided into independent (score = 6) and dependent (score ≤5) groups. Independent‐sample *t*‐tests were used to compare variables between groups, with significance set at *p* < .05. To determine which independent variables predicted basic ADL independence, a multivariate binary logistic regression analysis was performed using a significance level of *p* < .05 and 95% confidence intervals (CIs) to calculate odds ratios (ORs). Variables were entered into the regression model using the forward stepwise method, adjusted for age. All analyses were performed using SPSS version 20.0.

## 3 RESULTS

The sample comprised 231 individuals of both sexes aged 90 years and older (71% women). Anthropometric and clinical characteristics of the participants are shown in Table 1. Independent older adults showed better performance on the TUG test and higher MMSE scores, indicating a better cognitive status (*p* < .05).

**TABLE 1:** Characterisation, physical function and cognitive status data of the participants (*n* = 231).

	Independent (*n* = 119)	Dependent (*n* = 112)	*p*
Age (years)	93.2 ± 3.1	94.5 ± 3.6	.13
Body mass (kg)	64.8 ± 13.6	64.7 ± 13.5	.96
Height (m)	1.7 ± .1	1.6 ± .1	.06
HGS (kgf)	20.6 ± 6.7	16.2 ± 6.8	.74
TUG (s)	15.1 ± 12.9	23.3 ± 17.8	<.01
MMSE (pts)	23.9 ± 5.1	21.4 ± 6.8	<.01

Abbreviations: HGS, handgrip strength; MMSE, Mini‐Mental State Examination; TUG, Timed Up and Go Test.

Binary logistic regression adjusted for age was performed to determine which variables related to physical performance or cognitive function could predict independence in basic ADL. The results indicated that the model including TUG (OR = 4.3; 95% CI = 2.18–8.34) together with HGS (OR = 2.0; 95% CI = 1.14–3.45) provided the best fit [*χ* ^2^(2) = 30.82; *p* < .001; Nagelkerke *R* ^2^ = .167; −2 Log Likelihood = 289.20] (Table 2). This finding indicates that better performance on the TUG test increases the odds of an older person being independent by 4.3 times, and that each increase in HGS doubles these odds. Cognition assessed by the MMSE was not included in the regression model to explain independence in basic ADL (*p* > .05).

**TABLE 2:** Age‐adjusted binary logistic regression models of factors associated with independence in basic ADL (*n* = 231).

	*p*	OR_adjusted_	95% CI	−2 Log likelihood	*R* ^2^ de Nagelkerke
Model 1					
TUG	<.001	4.83	2.50–9.34	295.15	.136
Model 2					
HGS	.02	2.00	1.14–3.45	289.20	.167
TUG	<.001	4.27	2.18–8.34		

Abbreviations: HGS, handgrip strength; OR, odds ratio; TUG, Timed Up and Go Test.

## 4 DISCUSSION

This study aimed to compare physical function and cognitive status in nonagenarian and centenarian participants according to their level of dependence in basic ADL and to identify which parameters best predict functional independence. Although independent older participants exhibited superior physical performance and cognitive status in bivariate analyses, only physical function indicators remained independent predictors of the capacity to perform basic ADL.

In our study, shorter TUG times were associated with a 4.3‐fold greater likelihood of functional independence. Podsiadlo and Richardson (1991)^21^ observed that individuals completing the test in 20 s or less were generally independent, whereas those taking more than 30 s were dependent on ADL. Another study reported an incident disability probability of .13 over 2 years for those completing the TUG in 13 s, rising to .64 if the test took 20 s.^22^ It is important to consider that the TUG test assesses motor skills essential for daily activities, such as growing, walking and sitting, and also involves complex functional movements, such as transfers and turns, which are critical for instrumental ADL but also, to some extent, for basic tasks, such as toileting.^5^, ^19^ Thus, the TUG test is a robust marker of functional capacity in the oldest adults and is a valuable clinical screening tool for dependency risk.

Regarding muscle strength, Wang et al. (2020)^23^ conducted a review to determine whether muscular measures predict basic and instrumental ADL dependency in older populations. They found that muscle strength and physical performance were associated with developing basic and instrumental ADL dependence over follow‐up. Handgrip strength has been shown to predict increased functional limitations and is currently the most reliable measure of muscle function,^7^ which aligns with our finding that each increment in HGS doubles the odds of functional independence. In addition, HGS also moderates the association between cognitive functioning and functional independence.^24^

Although HGS alone should not be used to measure overall muscle strength in older adults,^25^ it can effectively predict deterioration in both basic and instrumental ADL, as well as outcomes, such as falls, mental health, hospitalisation and mortality.^26^, ^27^, ^28^ Additionally, HGS appears to be more closely related to ADL decline than muscle mass, which can be influenced by disease, muscle use and morphology.^29^ Handgrip strength declines progressively with age, with an inflection point at 50 years, when the annual decrease accelerates from .06 kg to .37 kg.^30^ This suggests that HGS reflects critical aspects of functional capacity, such as neuromuscular integrity and physiological reserve, which are determinants of ADL autonomy. Our OR of 2.0 for HGS supports this perspective, implying that accelerated strength loss after age 50 may be an early marker of dependency risk.

Population‐based studies have reported a paradoxical coexistence of improved survival and maintenance of basic ADL, even in cognitive and physical decline, as observed in Chinese cohorts followed by Zeng et al. (2017).^14^ Termed the ‘success‐related benefits’, this phenomenon suggests that physical performance declines may reflect fundamental ADL incapacity, whereas cognitive changes do not necessarily affect these basic functions.^31^ The HGS and TUG presence in our final model indicates that physical function, more than cognitive performance, plays a central role in preserving autonomy in long‐lived older individuals.

The exclusion of cognitive function assessed using the MMSE from the regression model may be explained by several factors. First, although both basic and instrumental ADL require mobility and cognitive engagement, since any task involving planning and execution towards a goal requires executive function, basic ADL are simpler, routine tasks that demand less cognitive complexity.^5^, ^31^ Hence, mild cognitive deficits, especially among the longest‐lived older adults, may not significantly impair basic ADL. Nonetheless, this does not diminish the importance of both physical and cognitive function in maintaining functional independence. One study comparing resistance exercise and resistance plus dual‐task training in cognitively impaired older individuals demonstrated that both interventions improved basic functional capacity; however, only the dual‐task group showed additional cognitive gains, likely because dual‐task exercises require greater executive control.^32^ Conversely, prior research has shown that the TUG test is independently associated with executive function, memory and processing speed,^33^ which may partly explain our results. A good TUG result may reflect adequate physical function and sufficient preserved cognitive abilities for daily tasks.

As a study limitation, the cross‐sectional design precludes causal inference between the outcomes, particularly in heterogeneous age groups such as nonagenarians and centenarians. Recruitment of this age bracket also poses methodological challenges and potential selection bias, as study participants tend to be healthier. Longitudinal studies are required to confirm these findings. In contrast, our study expands the evidence on the relationship between physical and cognitive function as predictors of functional independence in a population highly vulnerable to adverse events, which is a significant strength.

## 5 CONCLUSIONS

In summary, physical function is closely related to functional independence in nonagenarians and centenarians. Those with better physical function and greater muscle strength had higher odds of being independent in basic ADL. Our findings reinforce that intervention strategies focusing on physical function are essential for maintaining and preserving autonomy in basic ADL among long‐lived older individuals.

## FUNDING INFORMATION

This study received funding from the Fundação de Amparo à Pesquisa do Estado do Rio Grande do Sul.

## CONFLICT OF INTEREST STATEMENT

No conflicts of interest declared.

## Data Availability

The data that support the findings of this study are available from the corresponding author upon reasonable request.
